# Information complementarity: A new paradigm for decoding quantum incompatibility

**DOI:** 10.1038/srep14317

**Published:** 2015-09-22

**Authors:** Huangjun Zhu

**Affiliations:** 1 Perimeter Institute for Theoretical Physics, Waterloo, On N2L 2Y5, Canada

## Abstract

The existence of observables that are incompatible or not jointly measurable is a characteristic feature of quantum mechanics, which lies at the root of a number of nonclassical phenomena, such as uncertainty relations, wave—particle dual behavior, Bell-inequality violation, and contextuality. However, no intuitive criterion is available for determining the compatibility of even two (generalized) observables, despite the overarching importance of this problem and intensive efforts of many researchers. Here we introduce an information theoretic paradigm together with an intuitive geometric picture for decoding incompatible observables, starting from two simple ideas: Every observable can only provide limited information and information is monotonic under data processing. By virtue of quantum estimation theory, we introduce a family of universal criteria for detecting incompatible observables and a natural measure of incompatibility, which are applicable to arbitrary number of arbitrary observables. Based on this framework, we derive a family of universal measurement uncertainty relations, provide a simple information theoretic explanation of quantitative wave—particle duality, and offer new perspectives for understanding Bell nonlocality, contextuality, and quantum precision limit.

Observables that are incompatible or not jointly measurable play a fundamental role in quantum mechanics and quantum information science. Profound consequences of incompatible observables were realized soon after the inception of quantum theory by Heisenberg in the seminal paper^1^, from which originated the idea of uncertainty relations^2^,^3^. Around the same time, Bohr conceived the idea of the complementarity principle^4^. A vivid manifestation is the famous example of wave—particle duality^4^,^5^,^6^,^7^,^8^,^9^. In addition, incompatible observables are intimately connected to Bell nonlocality^10^,^11^,^12^, Einstein—Podolsky—Rosen (EPR) steering^13^,^14^,^15^, contextuality^16^,^17^,^18^,^19^, superdense coding^20^, etc. The implications of incompatibility have never been fully explored, as reflected in a recent heated debate on as well as resurgence of interest in measurement uncertainty and error-disturbance relations^2^,^21^,^22^,^23^.

Most existing literature on incompatible observables focus on two sharp observables (those represented by self-adjoint operators), partly due to the lack of a suitable tool for dealing with more observables or generalized observables (those described by probability operator measurements, also known as positive operator valued measures). With the advance of quantum information science and technologies, it is becoming increasingly important to consider more general situations. Detection and characterization of incompatible observables is thus of paramount importance. There exist a number of different notions characterizing the compatibility relations among quantum observables; prominent examples include commutativity, nondisturbance, joint measurability, and coexistence^24^,^25^. For sharp observables, all four notions are equivalent^26^. For generalized observables, however, all of them are inequivalent: observables satisfying a former relation also satisfy a latter relation but not vice versa in general^24^,^25^.

Among the four notions of compatibility mentioned above, joint measurability is distinguished by its close relation to Bell nonlocality^10^,^11^,^12^ and EPR steering^13^,^14^,^15^. In particular, a set of observables is not joint measurable if and only if it can be used to reveal EPR steering^14^,^15^. In the rest of this paper, we shall focus on the compatibility relations captured by the notion of joint measurability. Although the compatibility of a set of observables can be determined by semidefinite programming^27^, the computational complexity increases exponentially with the number of observables. In addition, existing algorithms provide little intuition as to why a set of observables is compatible or not. Actually, no intuitive criteria is known for determining the compatibility of even two generalized observables, except for a few special cases, such as two binary observables in the case of a qubit^9^,^28^,^29^,^30^,^31^. What is worse, most known criteria are derived with either brute force or ad hoc mathematical tricks, which offer little insight even if the conclusions are found. In this work we aim to change this situation.

In addition to the detection of incompatibility, quantification of incompatibility is also of paramount importance. Incompatibility measures are closely related to quantitative wave—particle duality relations^5^,^6^,^7^,^8^,^9^ and measurement uncertainty relations^2^,^32^. In this context, it is instructive to distinguish two different uncertainty relations concerning state preparations and measurements, respectively, as clarified in ref. 32. The traditional uncertainty relation, encoded in the Robertson inequality^33^, characterizes preparation uncertainty. Although this is well known as the Heisenberg uncertainty relation, it is different from the measurement uncertainty relation Heisenberg had in mind^1^,^32^. Also, most other uncertainty relations known in the literature belong to this type, including many entropic uncertainty relations^3^. By contrast, few works have studied measurement uncertainty relations for a long time; notable exceptions include refs 34,35. Recently, increasing attention has been directed to measurement uncertainty relations and incompatibility measures^2^,^21^,^22^,^23^,^36^. However, most works are tailored to deal with restricted scenarios, such as von Neumann observables or two generalized observables. More powerful tools are needed to deal with general settings.

In this work we propose a new paradigm for detecting and characterizing incompatible observables. Our framework is based on simple information theoretical ideas and quantum estimation theory^37^,^38^. The *Fisher information* underpinning our study turns out to be more effective than Shannon information in capturing the compatibility relations among different observables. In particular, we introduce a family of universal criteria for detecting incompatible observables and a natural measure of incompatibility, which are applicable to arbitrary number of arbitrary observables. Based on this framework, we derive a family of universal measurement uncertainty relations, which substantially improve over known uncertainty relations in terms of the scope of applicability. We also provide a simple information theoretic explanation of quantitative wave—particle duality and derive complementary relations for more than two complementary observables. In addition, our work offers new perspectives for understanding Bell nonlocality, EPR steering, contextuality, and quantum precision limit.

## Results

### Simple ideas

Our approach for detecting and characterizing incompatible observables is based on two simple information theoretic ideas: (1) every observable or measurement can only provide limited information and (2) information is monotonic under data processing. The joint observable of a set of observables is at least as informative as each marginal observable with respect to any reasonable information measure. A set of observables cannot be compatible if any hypothetical joint measurement would provide too much information. These ideas are general enough for dealing with arbitrary number of arbitrary observables. Furthermore, they are applicable not only to the quantum theory, but also to generalized probability theories^39^,^40^. For concreteness, however, we shall focus on the quantum theory.

Although information measures are not a priori unique, we find the Fisher information^41^ is a perfect choice for our purpose. Compared with Shannon information commonly employed in relevant studies, Fisher information is usually quantified by a matrix instead of a scalar and is more suitable in characterizing different information provided by different observables. In particular, Fisher information is more effective in capturing the information tradeoff among incompatible observables. In addition, many tools in quantum estimation theory^37^,^38^ can be applied to derive incompatibility criteria and measures in a systematic way instead of relying on ad hoc mathematical tricks, as is the case in most existing studies. Consequently, the incompatibility criteria and measures we derive are more intuitive and have a wider applicability.

Suppose the states of interest are parametrized by a set of parameters denoted collectively by *θ*. A measurement is determined by a family of probability distributions 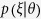 parametrized by *θ*. The Fisher information matrix associated with the measurement is given by


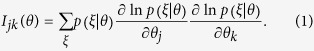


Its significance is reflected in the famous *Cramér*—*Rao bound*: the mean square error (MSE) matrix of any unbiased estimator of *θ* is bounded from below by the inverse Fisher information matrix (see supplementary information).

The set 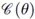 of Fisher information matrices *I*(*θ*) for all possible measurements is called the Fisher information *complementarity chamber* at *θ* for reasons that will become clear shortly. If there exists a unique maximal Fisher information matrix 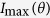, say, provided by the most informative measurement, as in the case of classical probability theory, then 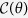 is represented by the intersection of two opposite cones characterized by the equation 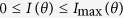. Except in the one-parameter setting, however, this is generally not the case for the quantum theory (and also generalized probability theories). Additional constraints on the complementarity chamber reflect subtle information tradeoff among incompatible observables, which is a direct manifestation of the complementarity principle. Alternatively, these constraints may be understood as epistemic restrictions imposed by the underlying theory.

### Characterize information complementarity with quantum estimation theory

To unleash the potential of the ideas presented in the previous section, it is essential to understand the structure of the complementarity chamber or, equivalently, the constraints on the set of realizable Fisher information matrices. In the case of quantum theory, a powerful tool for this purpose is quantum estimation theory developed over the past half century^37^,^38^,^42^,^43^ (see supplementary information).

A generalized observable or measurement is determined by a set of positive operators that sum up to the identity. Given a state 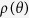 parametrized by *θ* and an observable 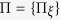, the probability of outcome *ξ* is given by the Born rule, that is, 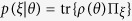. Accordingly, the Fisher information matrix takes on the form


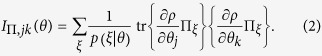


As mentioned previously, the inverse Fisher information matrix sets a lower bound for the MSE matrix of any unbiased estimator. However, the bound is applicable only to the specific measurement.

To understand the structure of the complementarity chamber, it is desirable to find constraints on the Fisher information matrix that is measurement independent. According to quantum estimation theory^37^,^38^,^42^,^43^, one important such constraint is the SLD (symmetric logarithmic derivative) bound 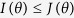, where 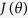 is the SLD quantum Fisher information matrix given by


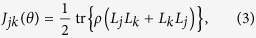


and *L*_*j*_ is the SLD associated with the parameter *θ*_*j*_ as determined by the equation


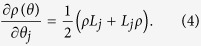


In the one-parameter setting, the SLD bound can be saturated by measuring the observable 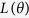; so the complementarity chamber 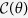 is a line segment determined by 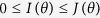. In the multiparameter setting, however, the bound generally cannot be saturated because the SLDs associated with different parameters are incompatible.

To determine the complementarity chamber in the multiparameter setting, it is necessary to consider additional constraints on the Fisher information matrix that take into account the information tradeoff among incompatible observables. Such information tradeoff is best characterized by the Gill—Massar (GM) inequality^42^


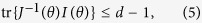


which is applicable to any measurement on a *d*-level system. To understand the significance of the GM inequality, note that the state space has dimension *d*^2^ − 1, so the upper bound in the above equation would be 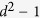 instead of *d* − 1 if the SLD bound can always be saturated. The GM inequality is useful not only to studying the complementarity chamber and compatibility problem but also to studying multiparameter quantum estimation problems^42^,^43^.

### Information complementarity illustrated

As an illustration, here we determine the complementarity chamber of the qubit in comparison with that of the probability simplex. In the case of a qubit, the GM inequality turns out to be both necessary and sufficient for characterizing the complementarity chamber. Moreover, any Fisher information matrix saturating the GM inequality can be realized by three mutually unbiased measurements^42^,^43^ (see supplementary information). This observation is crucial to attaining the tomographic precision limit in experiments^44^.

In terms of the components of the Bloch vector ***s***, the inverse quantum Fisher information matrix reads


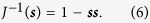


When *s* = 0 and thus *J* = 1, the complementarity chamber is a cone that is isomorphic to the state space of subnormalized states for the three-dimensional real Hilbert space, with its base (the set of Fisher information matrices saturating the GM inequality) corresponding to normalized states. Fisher information matrices of von Neumann measurements (determined by antipodal points on the Bloch sphere) correspond to normalized pure states, while those of noisy von Neumann measurements correspond to subnormalized pure states. When 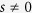, the complementarity chamber 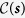 is a distorted cone. The *metric-adjusted complementarity chamber* 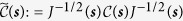, nevertheless, has the same size and shape irrespective of the parameter point.

**Figure 1:**
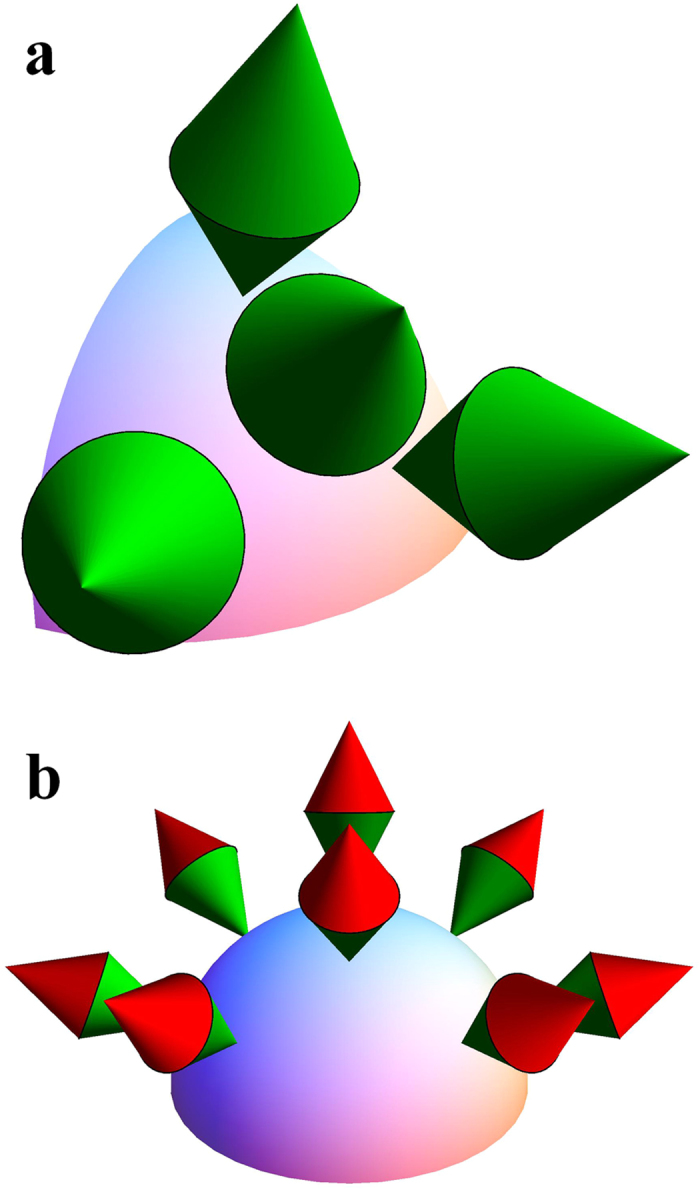
Metric-adjusted complementarity chambers. (**a**) Chambers (green cones, with modified size and aspect ratio for ease of viewing) on the probability simplex with respect to the Fisher—Rao metric^45^. (**b**) Chambers on the state space of the real qubit with respect to the quantum Fisher information metric. Each red cone represents the set of hypothetical Fisher information matrices satisfying the SLD bound but excluded by the GM inequality.

To visualize the complementarity chamber, it is instructive to consider the real qubit. With respect to the quantum Fisher information metric^45^, the state space is a hemisphere. Each metric-adjusted complementarity chamber is isomorphic to the state space for the two-dimensional real Hilbert space, and is represented by a circular cone, as illustrated in the lower plot of Fig. 1. This is in sharp contrast with the complementarity chamber on the probability simplex (with three components), which is represented by the union of two opposite cones; see the upper plot of Fig. 1. The missing cone of hypothetical Fisher information matrices for the real qubit is excluded by the GM inequality. Figure 1 is a vivid manifestation of the viewpoint that regards quantum theory as a classical probability theory with epistemic restrictions.

### Universal criteria for detecting incompatible observables

In this section we introduce a family of universal criteria for detecting incompatible observables, which are applicable to arbitrary number of arbitrary observables. So far we are not aware of any other criterion in the literature with such a wide scope of applicability. Our work fills an important gap on detecting incompatible observables and provides valuable insight on the joint measurement problem. In addition, our incompatibility criteria can be turned into criteria for detecting EPR steering given the close connection between the two subjects^14^,^15^; more details will be presented elsewhere.

Two (generalized) observables or measurements 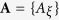 and 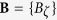 are *compatible* or jointly measurable if they admit a *joint observable* 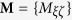, which satisfies


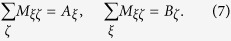


In that case, **A** and **B** are called *marginal observables* of **M**. Equivalently, **A** and **B** are compatible if they are coarse graining of a common observable 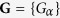, that is,


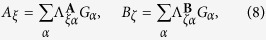


where 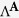 and 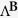 are two stochastic matrices^46^. Compatibility of more than two observables is defined similarly.

Suppose **M** is a joint observable of the set of observables **A**_*j*_; then 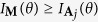 for any parameter point *θ* according to the Fisher information data-processing inequality^47^. Geometrically, this inequality means that 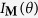 lies in the cone 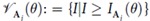 of hypothetical Fisher information matrices. If the **A**_*j*_ are compatible, then the intersection 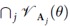 cannot be disjoint from the complementarity chamber 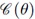. This constraint encodes a universal criterion on the compatibility of these observables.

A simpler compatibility criterion can be derived based on the observation that the Fisher information matrix *I*_M_ (*θ* is omitted for simplicity) needs to satisfy the GM inequality. Define 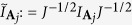 as the *metric-adjusted Fisher information matrix* and


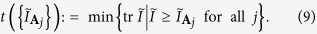


Then 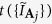 sets a lower bound for the GM trace 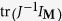 of any hypothetical joint observable **M** of observables **A**_*j*_. If the **A**_*j*_ are jointly measurable, then it must hold that


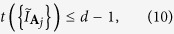


which yields a whole family of universal criteria for detecting incompatible observables upon varying the parameter point. These criteria are very easy to verify since 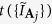 can be computed with semidefinite programming. The violation of the above inequality has a clear physical interpretation: Any hypothetical joint measurement of the **A**_*j*_ will enable estimating certain parameters with error at least 
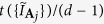
 times smaller than allowed by the quantum theory. To see this, let *I* be the Fisher information matrix provided by a hypothetical joint measurement **M** and 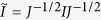. Then 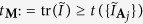. Setting 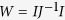 as the weighting matrix, then the GM bound for the weighted MSE of any unbiased estimator is given by 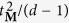 (see supplementary information). By contrast, the value achievable by the hypothetical joint measurement is 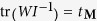, which is 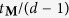 times smaller than the GM bound.

The function 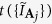 also enjoys one of two basic requirements for a good incompatibility measure, that is, monotonicity under coarse graining (see methods section). It is also unitarily invariant and thus may serve as a good incompatibility measure when the number of parameters under consideration is equal to the dimension *d*^2^ − 1 of the state space and the parameter point corresponds to the completely mixed state. This incompatibility measure, denoted by 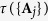 henceforth, can be expressed in a way that is manifestly parametrization independent and unitarily invariant (see supplementary information),


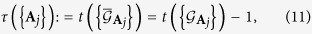


where 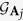 and 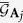 are metric-adjusted Fisher information matrices in superoperator form,


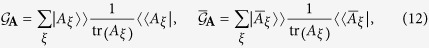


and 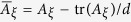. In the above equation, operators 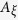 are taken as kets in the Hilbert-Schmidt space with the inner product 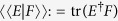; the double ket notation is adopted to distinguish operator kets from ordinary kets in the Hilbert space^43^,^48^. Superoperators, such as 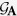, act on operator kets in the same way as operators act on ordinary kets. The threshold of the incompatibility measure 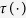 is *d* − 1. To reset the threshold when necessary, we may consider monotonic functions of *τ*, such as 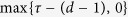 or 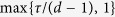.

### Universal measurement uncertainty relations

In this section we derive a family of universal measurement uncertainty relations, which are applicable to arbitrary number of arbitrary observables. As far as we know, no uncertainty relations with the same scope of applicability have been found before.

When a set of observables are incompatible, any approximate joint measurement entails certain degree of noisiness, which is a manifestation of measurement uncertainty relations^2^,^9^,^21^,^22^,^23^,^34^. A natural way of modeling noise on an observable, say 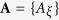, is coarse graining: 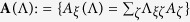, where Λ is a stochastic matrix characterizing the noise. Of particular interest is the type of coarse graining characterized by a single parameter: 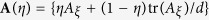 with 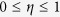. Coarse graining usually reduces the information gain; for example, 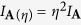 according to Eq. (2).

Suppose 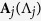 is a coarse graining of the observable **A**_*j*_ characterized by the stochastic matrix Λ_*j*_. Equation (10) applied to the 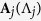 yields a family of universal uncertainty relations on the strengths of measurement noises,


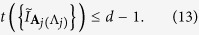


This equation means that to measure a set of incompatible observables approximately, individual observables must be noisy enough, so that they do not provide too much information than allowed by quantum mechanics. As far as we know, these measurement uncertainty relations are the only known examples that are applicable to arbitrary number of arbitrary observables. A special but important instance of Eq. (13) takes on the form


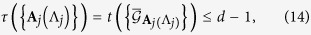


which is obtained when the number of parameters is equal to the dimension of the state space, and the parameter point corresponds to the completely mixed state. This equation reduces to 
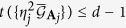
 when the noise on each observable **A**_*j*_ is characterized by a single parameter 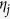, given that 
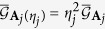
. If in addition all 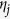 are equal to 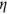, then we have 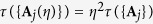, which leads to a simple measurement uncertainty relation,


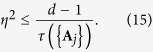


The incompatibility measure 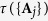 sets a lower bound for the amount of noise necessary for implementing an approximate joint measurement.

### Coexistence of qubit effects

To illustrate the power of our approach, here we consider the joint measurement problem of two noisy von Neumann observables 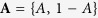 and 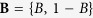 in the case of a qubit, where 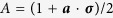 and 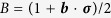. This problem is equivalent to the coexistence problem of the two effects *A* and *B*, which has attracted substantial attention recently^9^,^28^,^29^,^30^,^31^. Most known approaches rely on mathematical tricks tailored to this special scenario and allow no generalization. By contrast, our solution follows from a universal recipe based on simple information theoretic ideas.

According to Eq. (6), when *s* = 0, the quantum Fisher information matrix is equal to the identity. The Fisher information matrices of the two observables **A** and **B** are *I*_**A**_ = ***aa*** and *I*_**B**_ = ***bb***, respectively. Consequently,


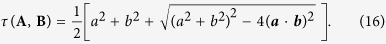


**Figure 2:**
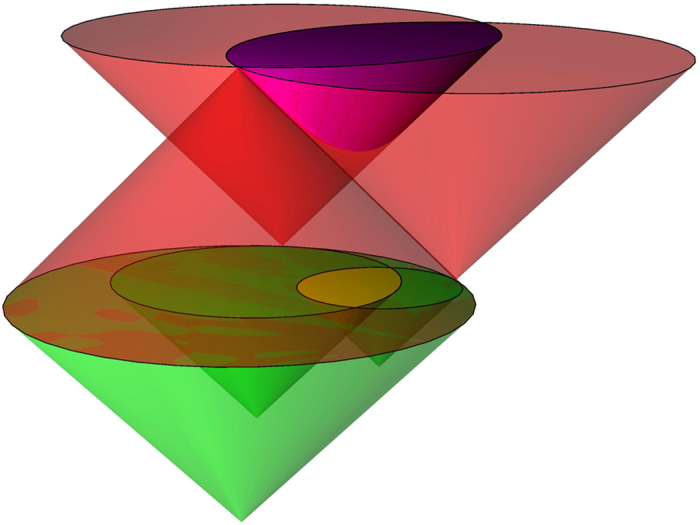
Information geometry of qubit observables. The largest green cone represents the complementarity chamber at the completely mixed state (cf. Fig. 1). The two upward red cones represent the sets of hypothetical Fisher information matrices lower bounded by the Fisher information matrices of two sharp von Neumann observables (corresponding to the tips of the cones), respectively. The two observables are incompatible since the intersection of the two cones is disjoint from the complementarity chamber. The distance from the intersection to the base of the complementarity chamber quantifies the degree of incompatibility. By contrast, their noisy versions corresponding to the tips of the two smaller green cones are compatible.

Remarkably, the inequality 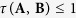 turns out to be both necessary and sufficient for the coexistence of 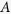 and 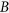. To verify this claim, it suffices to show its equivalence to the inequality 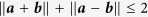 derived by Busch^28^, which is known to be both necessary and sufficient. Here the incompatibility measure *τ*(**A**, **B**) has a simple geometrical interpretation as the height (up to a scale) of the intersection 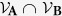 of two cones from the tip of the complementarity chamber, as illustrated in Fig. 2 (actually, this observation also offers a simple recipe for deriving *τ*(**A**, **B**). The inequality 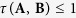 means that the intersection is not disjoint from the complementarity chamber. Otherwise, 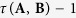 represents the distance from the intersection to the base of the chamber.

### Incompatibility of noncommuting sharp observables

It is well known that sharp observables are compatible if and only if they commute^26^. However, most known proofs rely on mathematical tricks without physical intuition. Here we reveal a simple information theoretic argument.

Commuting sharp observables are obviously compatible. To prove the converse, that is, compatible sharp observables commute with each other, it suffices to show that any observable **A** that refines a sharp observable **P** commutes with it. According to the Fisher information data-processing inequality^47^, 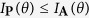, which implies that 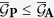 and 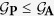 according to Sec. 3 in supplementary information, that is


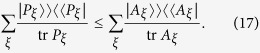


Taking inner product with 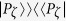 yields


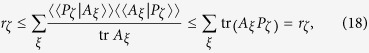


where 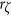 is the rank of 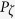. The inequalities are saturated if and only if each 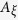 is supported either on the range of 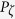 or on its orthogonal complement. Therefore, **A** commutes with **P**.

The degree of incompatibility of von Neumann observables (nondegenerate sharp observables) can be quantified by the measure *τ*, which turns out to be faithful now. Consider two such observables **A** and **B**, observing that 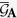 and 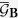 are rank-(*d* − 1) projectors, we have


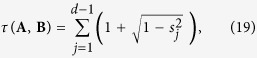


where the *s*_*j*_ are singular values of 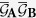 in decreasing order. The minimum *d* − 1 of 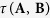 is attained when the first *d* − 1 singular values are all equal to 1, which amounts to the requirement 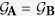, that is, **A** = **B**. The maximum 2(*d* − 1) is attained when all the singular values vanish, which happens if and only if **A** and **B** are complementary^49^.

Our approach also provides a universal measurement uncertainty relation between **A** and **B** as characterized by the inequality 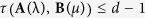, where


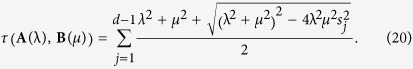


This inequality succinctly summarizes the information tradeoff between two von Neumann observables.

### Complementary observables and quantitative wave—particle duality

In this section we provide a simple information theoretic explanation of quantitative wave—particle duality and derive several complementary relations that are applicable to arbitrary number of complementary observables. Our study provides a natural framework for generalizing previous works specializing in the information tradeoff between two complementary observables associated with path information and fringe visibility, respectively^5^,^7^,^8^.

The complementarity principle states that quantum systems possess properties that are equally real but mutually exclusive^4^,^5^,^6^,^7^,^8^. In the quintessential example of the double-slit experiment, the photons (or electrons) may exhibit either particle behavior or wave behavior, but the sharpening of the particle behavior is necessarily accompanied by the blurring of the wave behavior, and vice versa. This wave—particle dual behavior is a manifestation of the impossibility of simultaneously measuring complementary observables^50^,^51^, say *σ*_*x*_ and *σ*_*z*_. Any attempt to acquire information about both observables is restricted by certain measurement uncertainty relation. For example, the two unsharp observables 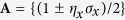 and 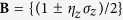 are jointly measurable if and only if^9^,^28^


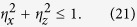


Coincidentally, this inequality is an immediate consequence of our general inequality 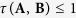 inspired by simple information theoretic ideas. Therefore, wave—particle duality can be understood as an epistemic restriction on the information content of observation.

Complementary relations, however, are not restricted to two observables. The potential of our approach lies in its capability in dealing with arbitrary number of observables. Suppose 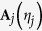 are unsharp versions of complementary observables 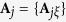. Then 
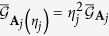
, where 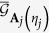 and 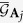 are metric-adjusted Fisher information superoperators. In addition, 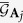 are mutually orthogonal rank-(*d* − 1) projectors. Therefore,


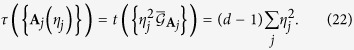


If the 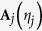 are jointly measurable, then the inequality 
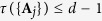
 generalizes Eq. (21) by setting a universal bound for the degree of unsharpness of these observables,


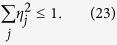


More generally, if the unsharpness of each observable **A**_*j*_ is characterized by a doubly stochastic matrix Λ_*j*_, then Eq. (22) generalizes to


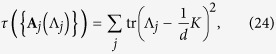


where *K* is the matrix with all entries equal to 1. Again, the inequality 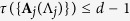 constrains the information tradeoff among complementary observables **A**_*j*_.

### Bell inequality

Our simple information theoretic ideas can also shed new light on Bell nonlocality^10^,^12^. As an illustration, here we show that given two observables for one party, the maximum violation of the CHSH inequality^11^ is a simple function of the measure of incompatibility introduced in this paper. Since Bell nonlocality may be seen as a special instance of contextuality^16^,^17^,^19^, our work is also helpful to this latter subject.

Suppose we have two ±1 valued observables *A* and *B* for party 1 together with similar observables *C* and *D* for party 2 (here we use Hermitian operators to represent observables following common convention; *A* is equivalent to **A** = {*A*_±_} in our convention, where *A*_±_ are the eigenprojectors of *A*). A bipartite state *ρ* satisfies the CHSH inequality if and only if 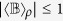
27,52, where


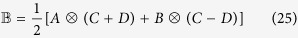


is the CHSH operator and satisfies


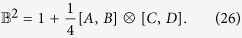


Given the observables *A* and *B* for party 1, the maximal violation of the CHSH inequality is attained when *C* and *D* are anticommuting Pauli matrices^27^,^52^,


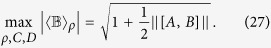


In the case party 1 is a qubit, suppose 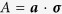 and 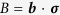 with unit vectors ***a*** and ***b***. Then


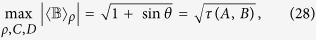


where *θ* is the angle spanned by vectors ***a*** and ***b***. Remarkably, the maximum is equal to the square root of the measure of incompatibility of *A* and *B* built on simple information theoretic ideas. This observation may have profound implications for understanding Bell inequalities from information theoretic perspectives.

In general, we can find spectral decompositions 
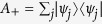
 and 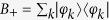 (which correspond to the singular value decomposition of *A*_+_*B*_+_) such that 
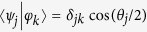
 with 0 ≤ *θ*_*j*_ ≤ *π*. Without loss of generality, we assume 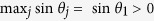. Then


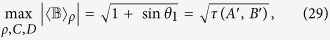


and the maximum is attained at a Bell state whose local support for party 1 is spanned by 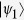 and 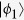. Here 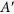 and 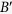 are the restrictions of *A* and *B* on this two-dimensional subspace.

## Summary

We have introduced a new paradigm for detecting and characterizing incompatible observables starting from two simple information theoretic ideas, quite in the spirit of the slogan “physics is informational”. Unlike most studies on this subject based on Shannon information, our work employs Fisher information to capture the information tradeoff among incompatible observables, which turns out to be surprisingly effective. This line of thinking is quite fruitful in studying a number of foundational issues. In particular, we introduced a family of universal criteria for detecting incompatible observables, which are applicable to arbitrary number of arbitrary observables. These criteria fill an important gap on detecting incompatible observables and provide valuable insight on the joint measurement problem. They are also useful for detecting EPR steering given the close connection between steering and incompatible observables. The same idea also leads to a natural measure of incompatibility, which can easily be computed by semidefinite programming. By virtue of this framework, we derived a family of universal measurement uncertainty relations, which are applicable to arbitrary number of arbitrary observables. In addition, our work provided a simple information theoretic explanation of quantitative wave—particle duality and offered new perspectives for understanding Bell nonlocality, contextuality, and quantum precision limit. Our study is of interest to researchers from diverse fields, such as information theory, quantum estimation theory, quantum metrology, and quantum foundations.

## Methods

### Measures of incompatibility

Here we discuss briefly how to quantify the degree of incompatibility of a set of observables, motivated by the discussions in the main text. To simplify the notation, we focus on two observables, say 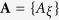 and 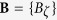; the generalization to more observables is immediate.

### Basic requirements

Like an entanglement measure, any good incompatibility measure, say *τ*(**A**, **B**), should satisfy certain basic requirements, among which the following two are very natural:

1. Unitary invariance: 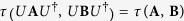;
2. Monotonicity under coarse graining.

Additional requirements, such as continuity, faithfulness, and choices of the scale and threshold may be imposed if necessary. To ensure great generality, however, we shall retain only the most basic requirements. Here the first requirement is self explaining. To make the second one more precise, we need to introduce an order relation on observables following Martens and de Muynck^34^.

Observable **C** is a *coarse graining* of **A** if 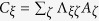 for some stochastic matrix 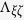, which satisfies 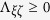 and 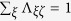. By contrast, we say **A** is a *refinement* of **C**. This order relation is denoted by 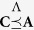 (or 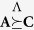), where the symbol Λ may be omitted if it is of no concern. It has a clear operational interpretation: Any setup that realizes the observable **A** can also realize **C** with suitable data processing as specified by the stochastic matrix. It is straightforward to verify that the order relation just defined is reflexive and transitive. Two observables **A** and **C** are *equivalent* if 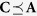 and 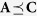. Such observables provide the same amount of information and may be identified if we are only concerned with their information contents. The resulting order relation on equivalent classes is antisymmetric in addition to being reflexive and transitive, and is thus a partial order.

Suppose four observables **A**, **B, C, D** satisfy 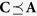 and 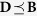. If **A** and **B** are compatible, then **C** and **D** are also compatible. Requirement 2 on the incompatibility measure amounts to the inequality 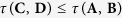, which may be seen as a natural extension of the above intuition.

### Robustness

A simple incompatibility measure can be defined in analogy with the entanglement measure robustness. Define 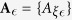 with


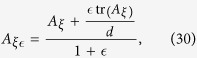


The *robustness R*(**A**, **B**) of two observables **A** and **B** is defined as the minimal nonnegative number 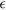 such that 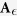 and 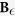 are compatible. A close relative of this measure is the logarithmic robustness 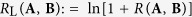. It is straightforward to verify that the robustness is unitarily invariant and faithful. To show monotonicity under coarse graining, note that 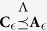 whenever 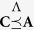. Suppose 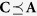 and 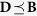; then 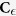 and 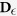 are compatible whenever 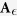 and 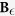 are. So 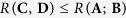; that is, the robustness is nonincreasing under coarse graining.

### Incompatibility measure inspired by quantum estimation theory

In this section, we introduce an incompatibility measure based on quantum estimation theory and simple information theoretic ideas presented in the main text. It is easy to compute and is useful for detecting and characterizing incompatible observables.

Our starting point is the observation that 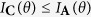 whenever 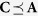, as follows from the Fisher information data-processing inequality^47^. In particular, the Fisher information has the nice property of being independent of representative observables in a given equivalent class. For example, it is invariant under relabeling of outcomes or “splitting” of an outcome, say 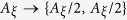, which has little physical significance. We note that few other information or uncertainty measures satisfy this natural requirement.

As an implication of the above analysis, 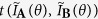 is monotonic under coarse graining, where 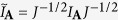 is the metric-adjusted Fisher information and 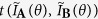 is defined in Eq. (9) in the main text. If the number of parameters is equal to *d*^2^ − 1, and the parameter point *θ* corresponds to the completely mixed state, then


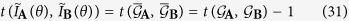


according to Sec. 3 in supplementary information, where 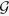 and 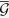 are metric-adjusted Fisher information superoperators as specified in Eq. (25) there and Eq. (12) in the main text. Define


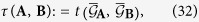


then *τ*(**A**, **B**) is both unitarily invariant and monotonic, thereby satisfying the two basic requirements of a good incompatibility measure. The threshold of *τ*(**A**, **B**) is *d* − 1. If 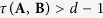, then the two observables **A** and **B** are necessarily incompatible; otherwise, either possibility may happen. To derive a measure with a usual threshold, we may opt for 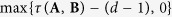 instead of *τ*(**A**, **B**). In this paper, however, we are mostly concerned with the ratio *τ*(**A**, **B**)/(*d* − 1).

Although *τ*(**A**, **B**) is generally not faithful, it provides lower bounds for the faithful measures *R*(**A**, **B**) and *R*_L_(**A**, **B**),


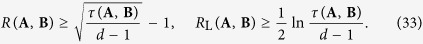


This equation is an immediate consequence of the observation 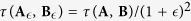. In addition, it is faithful on von Neumann observables, as demonstrated in the main text. These nice properties corroborate *τ* as a good incompatibility measure.

## Additional Information

**How to cite this article**: Zhu, H. Information complementarity: A new paradigm for decoding quantum incompatibility. *Sci. Rep.* **5**, 14317; doi: 10.1038/srep14317 (2015).

## Supplementary Material

Supplementary Information
