# Supplementary material for: Information complementarity: A new paradigm for decoding quantum incompatibility
Source: Sci Rep. 2015 Sep 22;5:14317. doi: 10.1038/srep14317 (PMC4585721; doi:10.1038/srep14317)
Supplement: Supplementary Information [file srep14317-s1.pdf]

Information complementarity: A new paradigm for decoding quantum incompatibility - Supplementary information

Huangjun Zhu

Perimeter Institute for Theoretical Physics, Waterloo, On N2L 2Y5, Canada

August 5, 2015

In this supplementary information we provide brief introduction on Fisher information, Cramér–Rao bound, and quantum estimation theory. The concepts of quantum Fisher information, symmetric logarithmic derivative (SLD), quantum Cramér–Rao bound, Gill–Massar inequality, and Gill Massar bound are reviewed. We also determine the complementarity chamber of the qubit based on the Gill–Massar inequality. In addition, we introduce parameter-free formulations of the SLD bound and the Gill–Massar inequality, which are useful to studying incompatibility criteria and measures.

1 Fisher information

The Fisher information [1] quantifies the amount of information provided by an observation or a measurement concerning certain parameters of interest. It determines the minimal error achievable in estimating these parameters through the Cramér–Rao bound [2, 3]. It is a basic tool in statistical inference and also plays crucial roles in various branches of physics and science in general [4, 5]. Here our interest in Fisher information stems from its potential applications in understanding a number of foundational issues in quantum mechanics, as presented in the main text.

Consider a family of probability distributions $p(\xi|\theta)$ parametrized by θ. Our task is to estimate the value of θ as accurately as possible based on the measurement outcomes. Given an outcome ξ, the probability $p(\xi|\theta)$ considered as a function of θ is called the *likelihood function*. The *score* is defined as the partial derivative of the log-likelihood function with respect to θ and reflects the sensitivity of the log-likelihood function with respect to the variation of θ. Its first moment is zero, and the second moment is known as the *Fisher information* [1],

$$I(\theta)=\sum_\xi p(\xi|\theta)\Bigl(\frac{\partial \ln p(\xi|\theta)}{\partial\theta}\Bigr)^2=\sum_\xi \frac{1}{p(\xi|\theta)}\Bigl(\frac{\partial p(\xi|\theta)}{\partial\theta}\Bigr)^2. \tag{1}$$

The Fisher information represents the average sensitivity of the log-likelihood function with respect to the variation of θ. Intuitively, the larger the Fisher information, the better we can estimate the value of the parameter θ.

An estimator $\hat{\theta}(\xi)$ of the parameter θ is *unbiased* if its expectation value is equal to the true parameter; that is,

$$\sum_\xi p(\xi|\theta)[\hat{\theta}(\xi)-\theta]=0. \tag{2}$$

In that case the variance or mean square error (MSE) of the estimator is lower bounded by the inverse of the Fisher information, which is known as the *Cramér–Rao bound* [2, 3].

In the multiparameter setting, the Fisher information takes on a matrix form,

$$I_{jk}(\theta) = \sum_{\xi} p(\xi|\theta) \frac{\partial \ln p(\xi|\theta)}{\partial \theta_j} \frac{\partial \ln p(\xi|\theta)}{\partial \theta_k}. \tag{3}$$

Accordingly, the Cramér–Rao bound for any unbiased estimator turns out to be a matrix inequality. Thanks to Fisher's theorem [6, 1], the lower bound can be saturated asymptotically with the maximum likelihood estimator under very general assumptions.

2 Quantum estimation theory

Here we give a short introduction to quantum estimation theory tailored to the needs in the main text. More details can be found in Refs. [7, 8, 9, 10].

In quantum parameter estimation, we are interested in the parameter that characterizes the state $\rho(\theta)$ of a quantum system. To estimate the value of this parameter, we may perform generalized measurements. Given a measurement Π with outcomes Π_ξ, the probability of obtaining the outcome ξ is $p(\xi|\theta) = \mathrm{tr}\{\rho(\theta)\Pi_\xi\}$. The corresponding Fisher information $I_\Pi(\theta)$ reads

$$I_\Pi(\theta) = \sum_{\xi} \frac{1}{p(\xi|\theta)} \mathrm{tr}\left\{\frac{\mathrm{d}\rho(\theta)}{\mathrm{d}\theta}\Pi_\xi\right\}^2. \tag{4}$$

Once a measurement is chosen, the inverse Fisher information sets a lower bound for the MSE of any unbiased estimator, which can be saturated asymptotically by the maximum likelihood estimator, as in the case of classical parameter estimation. It should be noted that the bound depends on the specific measurement.

2.1 Quantum Fisher information

A measurement independent bound for the MSE can be derived based on the *quantum Fisher information* [11, 7, 8]:

$$J(\theta) = \mathrm{tr}\{\rho(\theta)L(\theta)^2\}, \tag{5}$$

where $L(\theta)$ satisfies the equation

$$\frac{\mathrm{d}\rho(\theta)}{\mathrm{d}\theta} = \frac{1}{2}[\rho(\theta)L(\theta) + L(\theta)\rho(\theta)] \tag{6}$$

and is known as the *symmetric logarithmic derivative* (SLD) of $\rho(\theta)$ with respect to θ. The quantum Fisher information $J(\theta)$ is a an upper bound for the Fisher information $I(\theta)$, which is referred to as the SLD bound henceforth. The bound can be saturated by measuring the observable $L(\theta)$. Therefore, in the one-parameter setting, the complementarity chamber $\mathscr{C}(\theta)$ is a line segment determined by the equation $0 \leq I(\theta) \leq J(\theta)$. In conjunction with the classical Cramér–Rao bound, the inverse quantum Fisher information sets a lower bound for the MSE of any unbiased estimator, which is known as the quantum Cramér–Rao bound [11, 7, 8]. In this paper, we are more concerned with the SLD bound $I(\theta) \leq J(\theta)$ itself rather than the bound for the MSE.

In addition to its application in quantum estimation theory, the quantum Fisher information also plays an important role in studying the geometry of quantum states [12, 13, 14, 15]. For example, the SLD quantum Fisher information allows defining a statistical metric in the state space that is equal to four times of the Bures metric [12] and generalizes the Fisher–Rao metric defined on the probability simplex [1, 3, 15]. With respect to this metric, the Bloch ball is a 3-hemisphere. Also, the SLD quantum Fisher information plays a crucial role in studying parameter-based uncertainty relations [16].

In the multiparameter setting both the Fisher information and the quantum Fisher information take on matrix form,

$$\begin{aligned} I_{\Pi,jk}(\theta) &= \sum_\xi \frac{1}{p(\xi|\theta)} \operatorname{tr}\{\rho_{,j}\Pi_\xi\} \operatorname{tr}\{\rho_{,k}\Pi_\xi\}, \\ J_{jk}(\theta) &= \frac{1}{2} \operatorname{tr}\{\rho(L_j L_k + L_k L_j)\}, \end{aligned} \tag{7}$$

where $\rho_{,j} = \partial\rho(\theta)/\partial\theta_j$ and L_j is the SLD associated with the parameter θ_j. As in the one-parameter setting, $J(\theta)$ is an upper bound for $I(\theta)$. However, the bound generally cannot be saturated except when the L_j can be measured simultaneously. Consequently, the complementarity chamber is usually a small subset of the set of hypothetical Fisher information matrices satisfying the SLD bound. This difference is the main reason why multiparameter quantum estimation problems are so difficult and poorly understood. Surprisingly, however, this distinction can also be turned into a powerful tool for studying the complementarity principle, uncertainty relations and, in particular, the joint measurement problem, which are the focus of the main text.

2.2 Gill–Massar inequality

To better characterize the complementarity chamber in the multiparameter setting, we need more powerful tools than the SLD bound. One important tool is the following inequality derived by Gill and Massar [9] in the context of quantum state estimation,

$$\operatorname{tr}\{J^{-1}(\theta)I(\theta)\} \le d-1, \tag{8}$$

which is applicable to any measurement on a d-level system. The upper bound is saturated for any rank-one measurement when the number of parameters to be estimated is equal to the dimension d^2-1 of the state space. The Gill–Massar (GM) inequality succinctly summarizes the information trade-off among incompatible observables in multiparameter quantum estimation problems. It sets a lower bound for the weighted mean square error (WMSE) of any unbiased estimator [9, 10],

$$\mathcal{E}_W^{\mathrm{GM}} = \frac{\left(\operatorname{tr}\sqrt{J^{-1/2}WJ^{-1/2}}\right)^2}{d-1}, \tag{9}$$

where W is the weighting matrix (to simplify the notation we have omitted the dependence on the parameter θ). The lower bound can be saturated if and only if the hypothetical Fisher information matrix

$$I_W = (d-1)J^{1/2}\frac{\sqrt{J^{-1/2}WJ^{-1/2}}}{\operatorname{tr}\sqrt{J^{-1/2}WJ^{-1/2}}}J^{1/2} \tag{10}$$

belongs to the complementarity chamber. For example, the weighting matrix for the mean square Bures distance is equal to one fourth of the quantum Fisher information matrix, and the GM bound is $(d+1)^2(d-1)/4$. The bound can be saturated if and only if the complementarity chamber $\mathscr{C}$ contains $J/(d+1)$.

Both the Fisher information and quantum Fisher information depend on the parametrization of the state space; a judicial choice is often crucial to simplifying the discussion. For example, with a suitable parametrization, we can turn the quantum Fisher information matrix into the identity at least for a particular parameter point, say, $\tilde{\theta}$. Then the SLD bound and the GM inequality reduce to $I(\tilde{\theta}) \le 1$ and $\operatorname{tr}\{I(\tilde{\theta})\} \le d-1$, respectively.

2.3 Complementarity chamber for the qubit

In the case of a qubit, the GM bound for the WMSE can always be saturated, and the GM inequality is both necessary and sufficient for characterizing the complementarity chamber. Moreover, any

Fisher information matrix saturating the GM inequality can be realized by three mutually unbiased measurements. To verify this claim, note that the inverse quantum Fisher information matrix reads $J^{-1}(\boldsymbol{s}) = 1 - \boldsymbol{s}\boldsymbol{s}$ in terms of the components of the Bloch vector $\boldsymbol{s}$. Suppose that I_W in Eq. (10) has eigenvalues a_1, a_2, a_3 along with orthonormal eigenvectors $\boldsymbol{r}_1, \boldsymbol{r}_2, \boldsymbol{r}_3$. Denote by s_1, s_2, s_3 the three components of the Bloch vector in this basis. Then the GM bound can be saturated by measuring each observable $\sigma_j := \boldsymbol{r}_j \cdot \boldsymbol{\sigma}$ with probability $a_j(1 - s_j^2)$. Note that the probabilities are normalized since $\sum_j a_j(1 - s_j^2) = \operatorname{tr}(J^{-1} I_W) = 1$. Therefore, the desired measurement scheme can always be realized with a complete set of mutually unbiased measurements as claimed.

Alternatively, the structure of the complementarity chamber can be understood by analogy as in the main text. For simplicity, we shall focus on the parameter point $s = 0$; the general situation can be analyzed along the same line of thinking. Since $J = 1$ at $s = 0$, the set of Fisher information matrices saturating the GM inequality is isomorphic to the state space of the three-dimensional real Hilbert space. The extremal points of this set correspond to pure states, which form a real projective space of dimension two. Each extremal Fisher information matrix can be realized by a von Neumann measurement. A generic Fisher information matrix in this set can be expressed as a convex combination of three extremal Fisher information matrices, in analogy with the spectral decomposition of the corresponding state. Note that the von Neumann measurements realizing the three extremal Fisher information matrices are mutually unbiased. This observation confirms the same conclusion as in the previous paragraph. It should be noted that different convex decompositions of the given Fisher information matrix may lead to different realizations.

3 Parameter-free formulations of the SLD bound and the Gill–Massar inequality

The SLD bound and GM inequality can be formulated in a way that is parameter free [10]. Such formulations are often much easier to work with than the usual formulation and are quite useful in studying quantum estimation theory. They are particularly convenient to the current study since we are more interested in measurements rather than states. To derive such formulations, we need to recast the Fisher information matrix and quantum Fisher information matrix into superoperators.

3.1 SLD bound

Following the convention in Refs. [10, 17], the Hilbert–Schmidt inner product between two operators A and B is denoted by $\langle\!\langle A|B\rangle\!\rangle := \operatorname{tr}(A^\dagger B)$, where the double ket notation is used to distinguish them from ordinary kets. Given the state ρ and measurement Π, let $p_\xi = \operatorname{tr}(\rho\Pi_\xi)$ and $\bar{\Pi}_\xi = \Pi_\xi - \operatorname{tr}(\Pi_\xi)/d$. Let $\mathbf{I}$ denote the identity superoperator and $\bar{\mathbf{I}}$ the projector onto the space of traceless Hermitian operators. Define

$$\begin{aligned}\mathcal{F}(\rho) &:= \sum_\xi |\Pi_\xi\rangle\!\rangle \frac{1}{p_\xi} \langle\!\langle \Pi_\xi|,\\ \bar{\mathcal{F}}(\rho) &:= \bar{\mathbf{I}}\mathcal{F}(\rho)\bar{\mathbf{I}} = \sum_\xi |\bar{\Pi}_\xi\rangle\!\rangle \frac{1}{p_\xi} \langle\!\langle \bar{\Pi}_\xi|,\end{aligned} \tag{11}$$

where the dependence on Π is suppressed to simplify the notation. Then the Fisher information matrix can be written as

$$I_{jk}(\theta) = \langle\!\langle \rho_{,j}|\mathcal{F}(\rho)|\rho_{,k}\rangle\!\rangle = \langle\!\langle \rho_{,j}|\bar{\mathcal{F}}(\rho)|\rho_{,k}\rangle\!\rangle. \tag{12}$$

Therefore, $\bar{\mathcal{F}}(\rho)$ is essentially the Fisher information matrix in disguise [10, 17].

Define superoperator $\mathcal{R}(\rho)$ [12, 13, 14] by the equation

$$\mathcal{R}(\rho)|A\rangle\!\rangle = \frac{1}{2}|A\rho + \rho A\rangle\!\rangle. \tag{13}$$

Alternatively, $\mathcal{R}(\rho)$ can be written as

$$\mathcal{R}(\rho) = \frac{1}{2}\sum_{j,k=1}^{d}\big(|E_{jl}\rangle\rangle\rho_{jk}\langle\langle E_{kl}| + |E_{lk}\rangle\rangle\rho_{jk}\langle\langle E_{lj}|\big), \tag{14}$$

where the $E_{jk} := |j\rangle\langle k|$ form an operator basis. Define

$$\mathcal{J}(\rho) = \mathcal{R}^{-1}(\rho), \quad \bar{\mathcal{J}}(\rho) = \bar{\mathbf{I}}\mathcal{J}(\rho)\bar{\mathbf{I}}. \tag{15}$$

Then we have

$$J_{jk}(\theta) = \langle\langle\rho_{,j}|\mathcal{J}(\rho)|\rho_{,k}\rangle\rangle = \langle\langle\rho_{,j}|\bar{\mathcal{J}}(\rho)|\rho_{,k}\rangle\rangle. \tag{16}$$

Therefore, $\bar{\mathcal{J}}(\rho)$ is the superoperator analogy of the quantum Fisher information matrix.

Combining Eqs. (12) and (16), we recognize that the SLD bound for the Fisher information can be recast as

$$\bar{\mathcal{F}}(\rho) \le \bar{\mathcal{J}}(\rho). \tag{17}$$

3.2 Gill–Massar inequality

To derive alternative formulations of the GM inequality, we first note that the GM trace $\mathrm{tr}\{J^{-1}(\theta)I(\theta)\}$ is independent of the parametrization as long as the space spanned by the $\rho_{,j}$ is invariant. Let $\mathcal{P}$ be the projector onto this space, then

$$\mathrm{tr}\{J^{-1}(\theta)I(\theta)\} = \mathrm{Tr}\{[\mathcal{P}\mathcal{J}(\rho)\mathcal{P}]^{+}\mathcal{F}(\rho)\} = \mathrm{Tr}\{[\mathcal{P}\bar{\mathcal{J}}(\rho)\mathcal{P}]^{+}\bar{\mathcal{F}}(\rho)\}, \tag{18}$$

where A^{+} denotes the Moore-Penrose generalized inverse of A, which is equal to the inverse on the support of A when A is Hermitian. In addition, the GM trace is nondecreasing when the number of parameters increases or the space spanned by the $\rho_{,j}$ expands. Therefore,

$$\mathrm{tr}\{J^{-1}(\theta)I(\theta)\} \le \mathrm{Tr}\{\bar{\mathcal{J}}^{+}(\rho)\bar{\mathcal{F}}(\rho)\}, \tag{19}$$

where the inequality is saturated when the number of parameters is equal to d^2-1 or, equivalently, $\mathcal{P} = \bar{\mathbf{I}}$. Another crucial observation are the equalities

$$\langle\langle\rho|\mathcal{F}(\rho)|\rho\rangle\rangle = \sum_{\xi}\mathrm{tr}(\rho\Pi_{\xi}) = 1 \tag{20}$$

and

$$\bar{\mathcal{J}}^{+}(\rho) = \mathcal{J}^{-1}(\rho) - |\rho\rangle\rangle\langle\langle\rho|. \tag{21}$$

Consequently,

$$\mathrm{Tr}\{\bar{\mathcal{J}}^{+}(\rho)\bar{\mathcal{F}}(\rho)\} = \mathrm{Tr}\{\bar{\mathcal{J}}^{+}(\rho)\mathcal{F}(\rho)\} = \mathrm{Tr}\{\mathcal{J}^{-1}(\rho)\mathcal{F}(\rho)\} - 1, \tag{22}$$

Therefore, the GM inequality admits two equivalent formulations,

$$\mathrm{Tr}\{\bar{\mathcal{J}}^{+}(\rho)\bar{\mathcal{F}}(\rho)\} \le d-1, \quad \mathrm{Tr}\{\mathcal{J}^{-1}(\rho)\mathcal{F}(\rho)\} \le d. \tag{23}$$

The above formulations also lead to a much simpler proof of the GM inequality [10], whose original proof is quite convoluted.

$$\mathrm{Tr}\{\mathcal{J}^{-1}(\rho)\mathcal{F}(\rho)\} = \sum_{\xi}\frac{\langle\langle\Pi_{\xi}|\mathcal{J}^{-1}(\rho)|\Pi_{\xi}\rangle\rangle}{\langle\langle\rho|\Pi_{\xi}\rangle\rangle} = \sum_{\xi}\frac{\mathrm{tr}(\rho\Pi_{\xi}^{2})}{\mathrm{tr}(\rho\Pi_{\xi})} \le \sum_{\xi}\mathrm{tr}(\Pi_{\xi}) = d. \tag{24}$$

The inequality is saturated if the measurement is rank one.

In the case $\rho = 1/d$ and thus $\mathcal{R}(\rho) = \mathbf{I}/d$, the SLD bound in Eq. (17) reduces to $\bar{\mathcal{G}} \leq \bar{\mathbf{I}}$, where $\bar{\mathcal{G}}$ is the metric-adjusted Fisher information matrix in superoperator form, also known as the frame superoperator [18, 10, 17],

$$\bar{\mathcal{G}} := \bar{\mathbf{I}}\mathcal{G}\bar{\mathbf{I}}, \quad \mathcal{G} := \frac{1}{d}\mathcal{F}\Big(\frac{1}{d}\Big) = \sum_{\xi} |\Pi_\xi\rangle\rangle \frac{1}{\operatorname{tr}\Pi_\xi} \langle\langle \Pi_\xi|. \tag{25}$$

Accordingly, the GM inequalities in Eq. (23) reduce to

$$\operatorname{Tr}(\bar{\mathcal{G}}) \leq d-1, \quad \operatorname{Tr}(\mathcal{G}) \leq d. \tag{26}$$

In this special case, the GM inequalities are manifestly unitarily invariant.

References

[1] Fisher, R. A. Theory of statistical estimation. *Math. Proc. Cambr. Philos. Soc.* **22**, 700–725 (1925).

[2] Cramér, H. *Mathematical Methods of Statistics* (Princeton University Press, Princeton, NJ, 1946).

[3] Rao, C. R. Information and the accuracy attainable in the estimation of statistical parameters. *Bull. Calcutta Math. Soc.* **37**, 81–91 (1945).

[4] Frieden, B. R. *Physics from Fisher Information: A Unification* (Cambridge University Press, Cambridge, UK, 1999).

[5] Frieden, B. R. *Science from Fisher Information: A Unification* (Cambridge University Press, Cambridge, UK, 2004).

[6] Fisher, R. A. On the mathematical foundations of theoretical statistics. *Philos. Trans. R. Soc. Lond. A* **222**, 309–368 (1922).

[7] Helstrom, C. W. *Quantum Detection and Estimation Theory* (Academic Press, New York, 1976).

[8] Holevo, A. S. *Probabilistic and Statistical Aspects of Quantum Theory* (North-Holland, Amsterdam, 1982).

[9] Gill, R. D. & Massar, S. State estimation for large ensembles. *Phys. Rev. A* **61**, 042312 (2000).

[10] Zhu, H. *Quantum State Estimation and Symmetric Informationally Complete POMs.* Ph.D. thesis, National University of Singapore (2012). Available at `http://scholarbank.nus.edu.sg/bitstream/handle/10635/35247/ZhuHJthesis.pdf`. Date of access: 31/10/2012.

[11] Helstrom, C. W. Minimum mean-squared error of estimates in quantum statistics. *Phys. Lett. A* **25**, 101–102 (1967).

[12] Braunstein, S. L. & Caves, C. M. Statistical distance and the geometry of quantum states. *Phys. Rev. Lett.* **72**, 3439–3443 (1994).

[13] Petz, D. Monotone metrics on matrix spaces. *Linear Algebra Appl.* **244**, 81–96 (1996).

[14] Petz, D. & Sudár, C. Geometries of quantum states. *J. Math. Phys.* **37**, 2662–2673 (1996).

[15] Bengtsson, I. & Życzkowski, K. *Geometry of Quantum States: An Introduction to Quantum Entanglement* (Cambridge University Press, Cambridge, UK, 2006).

[16] Braunstein, S. L., Caves, C. M. & Milburn, G. J. Generalized uncertainty relations: Theory, examples, and Lorentz invariance. *Ann. Phys.* **247**, 135 – 173 (1996).

[17] Zhu, H. Quantum state estimation with informationally overcomplete measurements. *Phys. Rev. A* **90**, 012115 (2014).

[18] Zhu, H. & Englert, B.-G. Quantum state tomography with fully symmetric measurements and product measurements. *Phys. Rev. A* **84**, 022327 (2011).
